# Effect of telemedicine follow-up care of leg and foot ulcers: a systematic review

**DOI:** 10.1186/s12913-014-0565-6

**Published:** 2014-11-06

**Authors:** Lena Victoria Nordheim, Marianne Tveit Haavind, Marjolein M Iversen

**Affiliations:** Centre for Evidence-Based Practice, Bergen University College, Postbox 7030, N-5020 Bergen, Norway; Department of Medicine, Section of Endocrinology, Stavanger University Hospital, Stavanger, Norway

**Keywords:** Systematic review, Foot ulcers, Leg ulcers, Telemedicine

## Abstract

**Background:**

Leg ulcers and diabetes-related foot ulcers are frequent and costly complications of their underlying diseases and thus represent a critical issue for public health. Since the population is aging, the prevalence of these conditions will probably increase considerably and require more resources. Treatment of leg and foot ulcers often demands frequent contact with the health care system, may pose great burden on the patient, and involves follow-up in both primary and specialist care. Telemedicine provides potential for more effective care management of leg and foot ulcers. The objective of this systematic review of the literature was to assess the effect of telemedicine follow-up care on clinical, behavioral or organizational outcomes among patients with leg and foot ulcers.

**Methods:**

We searched Ovid MEDLINE (1980–), Ovid EMBASE (1980–), Clinical Trials in the Cochrane Library (via Wiley), Ebsco CINAHL with Fulltext (1981–) and SveMed + (1977–) up to May 2014 for relevant articles. We considered randomized controlled trials, non-randomized trials, controlled before-after studies and prospective cohort studies for inclusion and selected studies according to predefined criteria. Three reviewers independently assessed the included studies using the Cochrane Collaboration risk-of-bias tool. We performed a narrative synthesis of results and assessed the strength of evidence for each outcome using GRADE (grading of recommendations, assessment, development and evaluation).

**Results:**

Only one non-randomized study was included. The study (n = 140) measured the effect of real-time interactive video consultation compared with face-to-face follow-up on healing time, adjusted healing ratio and the number of ulcers at 12 weeks among patients with neuropathic forefoot ulcerations. There were no statistically significant differences in results of the different outcomes between patients receiving telemedicine and traditional follow-up. We assessed the study to have a high risk of bias.

**Conclusions:**

There is insufficient evidence available to unambiguously determine whether telemedicine consultation of leg and foot ulcers is as effective as traditional follow-up.

## Background

Leg ulcers and diabetes-related foot ulcers represent challenges for individual people and the health care system. Leg and foot ulcers are longstanding and costly complications of their underlying diseases and thus represent a critical issue for public health [1]. Since the population is aging, the prevalence of these conditions will probably increase considerably and require more resources [2]. The prevalence of leg ulcers is estimated to be 1.2–3.2% [3]. The annual incidence rate for diabetes-related foot ulcers varies from 1.2%–3.0% [4-6]. An increase in the proportion of older people will affect the need for health care, including the need for treatment and follow-up care of leg and foot ulcers. Although Norway is sparsely populated, with many rural areas, one national target is that health care services be provided as close as possible to the patients’ homes [1,7]. Information and communication technology (ICT) may contribute to achieving this target [2,8].

Telemedicine is a key part of the ICT and is used to achieve integrated health care services in Norway and is defined as “the use of electronic information and communication technologies to provide and support health care when distance separates the participants” [9]. Telemedicine solutions may contribute to improving local health care and to reducing patients’ burden related to traveling to and from treatment venues.

Treatment of leg ulcers and diabetic foot ulcers often demands frequent contact with the health care system and may pose a great burden on the patient [10-12]. Treating diabetic foot ulcers is particularly challenging to health care professionals, because the ulcer might take months to heal, can lead to osteomyelitis, gangrene and amputation [12]. Although leg ulcers patients may not experience the same severe consequences, incorrect implementation of treatment can delay healing and cause pain and trauma [13].

According to international guidelines patients with diabetic foot ulcers should be referred to specialist foot clinics at an early stage [14]. However, in Norway as well as other European countries many foot ulcer patients are treated a substantial time in primary care with lack of expert nurses and doctors and access to specialist health care [10,11,15]. Similarly, patients with leg ulcers are treated to a large extent by primary care nurses, which may be problematic as they may not be using the evidence base sufficiently well to support ulcer healing and patient well-being [13]. A critical point in ulcer care seems to be capacity problems in the specialist health care system and communication problems between primary care and specialist care [11,16].

An additional challenge in countries with many rural areas, such as Norway, is the follow up of these vulnerable patients in specialist health care as they are deemed to have a substantial travel time. The use of telemedicine provides a potential for a more effective management of this patient group due to a more active cooperation between primary and specialist health care, and quicker access to specialist health care when required [17]. Telemedicine follow-up might be an alternative to the current organization of specialist health care to realize the goal of coordinating and integrating care. This may provide positive health gains for patients by reducing travel time and increase their satisfaction with health care.

Telemedicine has been used in health care services for several years, both within disciplines (such as radiology and dermatology), for disease groups (such as diseases of the circulatory and respiratory systems) and for specific diseases such as diabetes [9,18-26]. Previous systematic reviews of the use of telemedicine services for various patient groups included people with diabetes, but not patients with leg and foot ulcers specifically [9,18-26]. One review indicated that telemonitoring improves health care, and documented how telemedicine affects clinical behavioral or organizational outcomes. However, studies involving healthcare providers in the capture and transmission of clinical patient data were not included [26]. Other systematic reviews concluded that the technology is user-friendly and that the quality of the images is adequate for diagnostic purposes [22,24-26]. For more effective management of ulcer care, telemedicine has been suggested as a solution to improve coordination between the different levels of care and to enhance the quality of care in the health care services [26]. However, the effectiveness of telemedicine interventions for patients with leg and foot ulcers regarding clinical, behavioral or organizational outcomes compared with traditional follow-up care is unclear. Health care personnel express a request for telemedicine follow up. Therefore from a clinical and research perspective there is a need to summarize the literature in a systematic review to consider whether telemedicine is adequate to provide appropriate follow-up care of leg and foot ulcers [18,27].

This review assesses whether telemedicine follow-up care of patients with leg and foot ulcers, specifically the transfer of digital still images or video consultations, affects clinical, behavioral or organizational outcomes compared with traditional follow-up care.

## Methods

### Criteria for inclusion and exclusion of studies

#### *Participants*

The review examined studies in which patients with arterial, venous and/or diabetes-related foot and/or leg ulcers participated. We considered studies including other types of ulcers if the authors provided separate results for the various types of ulcers.

#### *Intervention*

Transfer of digital still pictures or video consultation.

#### Comparison

Traditional face-to-face follow-up care (hereafter referred to as traditional follow-up).

#### *Outcomes*

At least one of the following three main groups of outcomes had to be reported: clinical outcomes (e.g. healing time of the ulcers); behavioral outcomes (e.g. change in degree of self-care or change in interaction between patient and health personnel); or organizational outcomes (e.g. change in interaction and/or cooperation between health personnel).

### Study design

We reviewed randomized controlled trials, non-randomized trials, controlled before-after studies and prospective cohort studies with a comparison of treated and non-treated groups. Studies published in English, Norwegian, Swedish or Danish were included. We searched for studies published from 1980 and onwards because relevant telemedical equipment was not available before 1980. We made no attempt to identify grey literature. The protocol is not registered on PROSPERO but is available from MTH on request (in Norwegian).

### Search strategy

We performed searches in Ovid MEDLINE (1980–) Ovid EMBASE (1980–), Clinical Trials in the Cochrane Library (via Wiley), Ebsco CINAHL with Fulltext (1981–) and SveMed + (1977–). The first searches were performed in October 2011, while an updated search was performed on May 16^th^ 2014. We developed a search strategy for Ovid MEDLINE and adapted it for the other databases (Table 1). Key search words were “telemedicine”, “video consultation”, “telephone”, “leg ulcer”, “foot ulcer” and “diabetic foot”. We reviewed the reference lists of studies for which we obtained full-text articles and other relevant articles. Experts in the field were contacted to identify unpublished or ongoing studies. We also performed a search for ongoing studies in ClinicalTrials.gov, Current Controlled Trials and Health Services Research Projects in Progress (HSRProj) on June 10^th^ 2014. We did not hand-search key journals.

**Table 1:** **Search strategy for Ovid MEDLINE**

1.	Telemedicine/
2.	Telecommunications/
3.	Electronic mail/
4.	Satellite communications/
5.	Remote consultation/
6.	Telephone/
7.	Cellular phone/
8.	Modems/
9.	Television/
10.	Videoconferencing/
11.	Video recording/
12.	Webcasts as topic/
13.	Wireless technology/
14.	exp Computer communication networks/
15.	or/1-14
16.	tele*.tw.
17.	(e-mail* or electronic mail*).tw.
18.	(ehealth* or e-health*).tw.
19.	(e-medicine* or emedicine*).tw.
20.	(videoconferen* or video-conferen*).tw.
21.	(videophone* or video-phone*).tw.
22.	medical record system*.tw.
23.	((mobile* or phone* or telephone*) adj3 (consult* or counsel*)).tw.
24.	((mobile* or phone* or telephone*) adj3 (follow up* or support* or interview*)).tw.
25.	(distan* adj4 (health* or consult* or counsel* or monitor*or treatment*)).tw.
26.	(remote* adj4 (health* or consult* or counsel* or monitor* or treatment*)).tw.
27.	image trans*.tw.
28.	picture trans*.tw.
29.	or/16-28
30.	or/15 or 29
31.	exp Leg ulcer/
32.	((leg or crural or cruris or venous or varicose or stasis or foot or plantar or sole or plantaris or pedis) adj2 (ulc* or sore* or wound*)).tw.
33.	(diabet* adj2 (foot* or feet* or ulc* or sore* or wound*)).tw.
34.	or/31-33
35.	30 and 34

*The asterisk (*) was used for truncation to search multiple forms of a free-text term (singular/plural, variable spellings, etc.), e.g. “ulc*” to find “ulcer”, “ulcers”, “ulcus”.

### Study selection

MTH and MMI independently screened all titles and abstracts identified through the first literature search, while LVN and MMI did the same for the final search. We obtained the full text of articles for all references identified as potentially meeting the inclusion criteria, and also in cases of uncertainty or when there were discrepancies between the reviewers during the screening process. LVN, MTH and MMI independently read all full-text articles followed by discussion to reach consensus.

We contacted the main author of five studies [28-32] by e-mail to obtain additional information about the studies. Four of the studies did not report separate outcome data for ulcer types relevant to the current review. Of these, three authors confirmed that separate data were not available [28,29] or that they did not have the capacity to extract the data [30]. The fourth author provided an incomplete response with regard to separate outcome data. However, she confirmed that telemedicine was used for diagnostic rather than follow-up purposes for the majority of patients included in the study [31]. Consequently, we excluded these four studies [28-31]. We contacted the fifth author because of uncertainty about a subgroup in the study [32]. The author clarified the issue and eventually the study was excluded.

### Data collection

We developed a data extraction form to record relevant study characteristics: study design, population characteristics, intervention characteristics, outcome characteristics and results in included studies.

### Assessment of risk of bias

LVN, MTH and MMI independently assessed risk of bias in the included studies by using a translated version of the Cochrane Collaboration risk-of-bias tool [33]. In regard to the tool’s last item (other potential biases), we specifically assessed potential confounding factors such as previous ulcers, the duration of the ulcers before treatment started, the extent of the ulcer, blood glucose control, malnutrition and mental health [34-39].

### Synthesis of the results and quality assessment

We did not perform a meta-analysis as only one study fulfilled the inclusion criteria. Instead, we performed a narrative summary of outcomes presented in the included study and assessed the strength of the evidence for each outcome using the GRADE version 3.6 (grading of recommendations, assessment, development and evaluation) approach to reviewing evidence [40].

## Results

**Figure 1:**
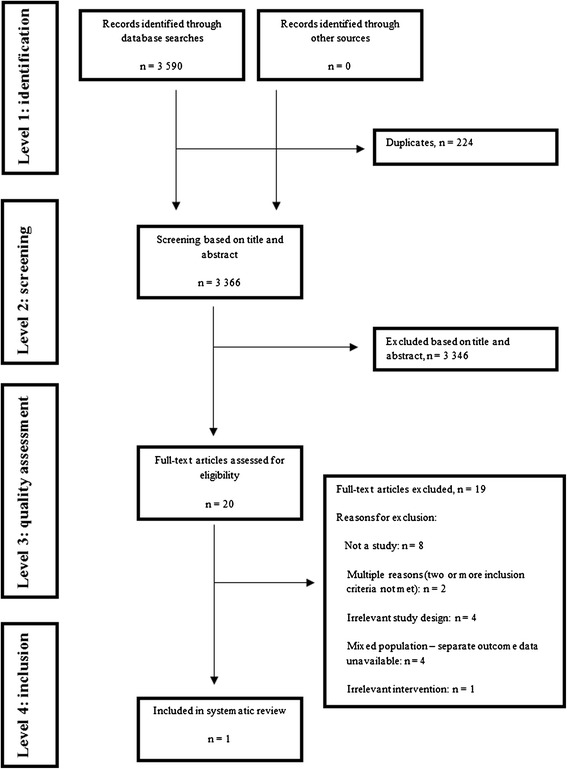
**Flow chart of study selection process.**

Electronic searches resulted in 3590 citations. Of these, 224 duplicates were removed and 3346 citations were excluded after reviewing the titles and abstracts. We obtained and read the full text of the remaining 20 citations. We excluded 19 citations due to the following reasons: not a study (n = 8), multiple reasons for exclusion (i.e. more than one inclusion criteria not met; n = 2), irrelevant study design (n = 4), mixed population and separate outcome data not available (n = 4), and irrelevant intervention (n = 1). Details for excluded studies in all but the first exclusion category are provided in Table 2 [28-32,41-46]. Contact with experts did not identify further studies. Eventually, only one study [47] was included in the review (Figure 1).

**Table 2:** **Characteristics of excluded studies**

**Study**	**Reason for exclusion**
Bowles 2002 [28]	Population: Diabetes patients with and without foot ulcers. Separate data for foot ulcers not available.
Dobke 2008 [30]	Population: Mixed, including patients with pressure ulcers. Separate data for venous/arterial leg ulcers and diabetic foot ulcers not available.
Edmondson 2010 [41]	Study design: uncontrolled before-after study
Edwards 2009 [42]	Intervention: not telemedicine follow-up
Hands 2006 [31]	Population: Mixed, separate data for venous/arterial leg ulcers and diabetic foot ulcers not provided by author. Author confirmed that telemedicine was used for diagnostic purposes rather than follow-up in the majority of patients.
Kim 2004 [32]	Study design: Prospective cohort study without comparison of exposed (telemedicine follow-up) and non-exposed (no telemedicine follow-up) patients.
Lazzarini 2010 [43]	Study design: Multiple case study
Manuel 2012 [44]	Study design: Uncontrolled before-after study
Nagykaldi 2003 [45]	Study design: Uncontrolled before-after study
	Population: Diabetes patients. Separate data for ulcers not available.
Nyheim 2010 [46]	Study design: Qualitative study
	Outcomes: Change in knowledge about chronic ulcers among nurses.
Santamaria 2004 [29]	Population: Mixed, including pressure ulcers and surgical ulcers. Separate data for foot and leg ulcer patients not available.

The search for ongoing studies resulted in three titles and projects that might be included in a future systematic review regarding the effect of telemedicine follow-up of foot and leg ulcers (Table 3).

**Table 3:** **Ongoing trials likely to meet inclusions criteria**

**Study/source**	**Country**	**Study design**	**Population**	**Treatment**	**Trial start/likely completion**
Clin. Trials.gov NCT01608425	Denmark	Randomized study	People with diabetes-related foot ulcers	Telemedicine consultations between ulcer-nurses in the primary sector and the wound clinics at the hospitals in the region.	2011/2013
Clin. Trials.gov NCT01710774	Norway	Cluster randomized study (non-inferiority)	People >20 years with diabetes-related foot ulcers enrolled in specialist health care	Telemedicine follow-up care in municipal primary health care in collaboration with specialist health care	2012/2016
Clin. Trials.gov NCT01814267	France	Randomized study	People with diabetes-related foot ulcers ≥18 years enrolled in specialist health care	Telemedicine care and follow-up in specialist health care	2013/2015

### Study characteristics

Table 4 gives an overview of the characteristics of the included study. The study was a non-randomized study conducted in the United States and comprised 140 people with diabetes-related foot ulcers. The purpose of the study was to compare the effectiveness of telemedicine follow-up of forefoot ulcerations with traditional face-to-face follow-up with regard to healing time [47].

**Table 4:** **Characteristics of the included study**

**Study, year (country)**	**Design**	**Setting**	**Study population**	**Intervention group**	**Control group**	**Outcomes**	**Results**
Wilbright, 2004 [47]	Non-randomized study	Two local medical centers located 55 miles apart	Total: 140 patients	Real time interactive video consultation, with or without transfer of digital images	Face-to-face follow-up in a specialized diabetes-related foot program	Healing time in days, percentage of ulcer healed after 12 weeks and healing time ratio adjusted for age, ulcer duration (days), location, size, crossover and severity grade	Average healing time in days: Intervention group = 43.2 ± 29.3
(USA)			Intervention group: 20 patients (55% women, average age 55.1 years)				Control group = 45.5 ± 43.4
							*P* = 0.83
			Control group: 120 patients (45% women, average age 56.5 years)				Adjusted ratio for healing time: Intervention group = 1.00
							Control group = 1.40
							*P* = 0.10
							Percentage of ulcers healed at 12 weeks: Intervention group = 75%
							Control group = 81%
							*P* = 0.55
							Not healed or lost to follow-up: Intervention group: 3/20
							Control group: 7/120
							No patient adverse effects were reported.

#### Participants

The study included 140 consecutive patients treated for neuropathic forefoot ulcerations from two medical centers. The patients from one center, the intervention group, comprised 20 patients treated via telemedicine consultation (55% women, average age 55.1 years). The other center, the control group, comprised 120 patients receiving traditional follow-up (45% women, average age 56.5 years).

#### Intervention

The intervention group received real-time interactive video consultation, with or without transfer of digital images of forefoot ulcerations, with a specialist nurse, physician and physiotherapist based at a remote medical center. The patients in the control group were treated face-to-face according to a specialized diabetes-related foot program at a local medical center. Both groups were given a standard follow-up program including routine follow-up, screening of the feet, lectures, guidance and adaptation of footwear [47]. The number of consultations per person for the intervention group or the control group was not stated. The patients were followed up for 12 weeks.

#### Outcome

The main outcomes were forefoot ulcer healing time in days, the percentage of ulcers healed after 12 weeks and healing time ratio adjusted for age, ulcer duration (days) location, size, crossover and severity grade. The authors did not report any adverse events or stated whether they assessed adverse events related to telemedicine follow-up.

### Risk of bias

We consider the risk of bias in the study to be high (Table 5), mainly due to the lack of randomization of participants to the intervention and control group. The participants and health personnel could not be blinded to the type of treatment received. In addition, the article does not state whether the outcome assessors were independent and blinded [47].

**Table 5:** **Assessment of risk of bias in the included study**

**Domain**	**Study**
	Wilbright et. al. [47]
**Was the allocation sequence adequately generated?**	No
**Was allocation adequately concealed?**	No
**Was knowledge of the allocated interventions adequately prevented by participants and personnel during the study?**	No
**Was knowledge of the allocated interventions adequately prevented by outcome assessors during the study?**	Unclear
**Were incomplete outcome data adequately addressed?**	No
**Are reports of the study free of suggestion of selective outcome reporting?**	No
**Was the study apparently free of other problems that could put it at a risk of bias?**	Yes
**Overall risk for bias**	High risk

Unclear = the risk of bias is unknown, or not relevant to the study. No = high risk of bias. Yes = low risk of bias.

The number of patients who either did not heal or were lost to follow-up was 3 of 20 (15%) in the telemedicine group and 7 of 120 (5%) in the control group. The researchers analyzed the healing time for dropout patients as censored events at 12 weeks of follow-up. It is unclear how the researchers analyzed patients who did not heal at 12 weeks.

The researchers made corrections for important confounders, for example healing time ratio was adjusted for age, ulcer severity, ulcer duration, location and size. The small sample size might not allow adjusting for other potential confounding factors.

### Study results

The unadjusted forefoot healing time for the telemedicine group and the control group: 43.2 ± 29.3 days for the telemedicine group versus 45.5 ± 43.4 days for the control group (*P* = 0.83) did not statistically significant differ. After adjusting for age, ulcer duration, location, size, crossover and severity grade, the intervention group and the control group did not statistically significant differ in healing ratio (1.40 versus 1.00, *P* = 0.10). Moreover, there were no statistically significant differences between groups in the number of ulcers healed at 12 weeks: 75% in the intervention group and 81% in the control group (*P* = 0.55) [47].

Using GRADE, we consider the strength of the evidence to be very low for all outcomes (Table 6). A high risk of bias due to limitations in the study design was the main reason why the study achieved a very low GRADE score.

**Table 6:** **GRADE assessment of the efficacy of video consultation of patients with leg and foot ulcers**

**Number of participants (study)**	**Outcome**	**Comparison**	**Study design**	**Quality assessment (risk of bias)**	**Consistency**	**Directness**	**Precision**	**Reporting bias**	**Result**	**GRADE assessment**	**Comments**
140 [47]	Unadjusted healing time (number of days)	Traditional consultation with diabetes-related foot team	2	−2	0	0	−2	0	Intervention group: 43.5 ± 29.3	Very low	Degraded because of the study design, high risk of bias and uncertain estimate of effectiveness
									Control group: 45.5 ± 43.4		
									*P* =0.83		
140 [47]	Adjusted healing time	Traditional consultation with diabetes-related foot team	2	−2	0	0	−2	0	Intervention group: 1.40	Very low	Degraded because of the study design, high risk of bias and uncertain estimate of effectiveness
									Control group: 1.00		
									*P* = 0.10		
140 [47]	Ulcers healed at 12 weeks	Traditional consultation with diabetes-related foot team	2	−2	0	0	−2	0	Intervention group: 75%	Very low	Degraded because of the study design, high risk of bias and uncertain estimate of effectiveness
									Control group: 81%		
									*P* =0.55		

## Discussion

To our knowledge this is the first systematic review with the purpose of summarizing studies measuring the effectiveness of telemedicine follow-up care of patients with leg and foot ulcers. The evidence that we identified [47] still renders it inconclusive whether telemedicine management of people with diabetes-related foot ulcers may be an equivalent alternative to traditional follow-up concerning the healing time of the ulcers. The strength of the evidence is very low and limited by the study design: a high risk of systematic bias, insufficient and partly inadequate reporting of predefined outcome values and few participants especially in the intervention group.

Randomized controlled trials with larger samples and sufficient follow-up time are needed to produce valid evidence about the effectiveness of telemedicine follow-up care of leg and foot ulcers. Even though health care services have used telemedicine for several years, studies that evaluate the effect of telemedicine have been limited to small-scale studies conducted over short periods of time [19]. Telemedicine interventions are complex and require considerable resources to carry out. Moreover, change in health care systems is slow and needs time to adapt and adopt new technologies. Thus, the time frame employed by Wilbright et al. [47], and other researchers [19] may have been too short to enable telemedicine technology to be adopted in the study settings and to evaluate effectiveness in randomized controlled trials.

A telemedicine intervention in patients with leg and foot ulcers can be considered to be a “complex intervention” as several components interact within the experimental and control group [48]. Challenges in developing, evaluating and synthesizing complex telemedicine interventions are for example the number of nurses involved in patient care and the different behaviors by those delivering the intervention. Telemedicine interventions also target at least two organizational levels, including primary health care and specialist health care. A strict standardization of telemedicine interventions may thus prove difficult and the intervention may be challenging to replicate and generalize across settings and studies. Accordingly, authors should carefully describe all components of the intervention when reporting future randomized studies. Furthermore, future intervention studies should standardize outcome measures [19,49], although defining explicit outcome criteria for treating ulcers is a challenge due to aetiology of wounds and multiple ways of assessing improvement, including wound-healing related outcomes (wound closure, reduction rate and healing time) and change in wound condition [49]. Adding to this challenge is the fact that patients with diabetes foot ulcers are fragile, have a relatively high age, comorbidites, and excess mortality [50]. Altogether these factors may explain why studies are lacking that evaluate the effectiveness of telemedicine follow-up of patients with leg and foot ulcers.

When randomizing patients at an individual level the same health professionals will treat patients in the intervention group and control group. This may threaten the validity of the study. Thus, in future research, there is a rationale for choosing cluster randomized trials where units such as geographical areas or institutions, are allocated to the different groups, [51]. A cluster randomized trial will therefore require a larger sample size as it will have to take into account dependency in data. Furthermore, an equivalence or non-inferiority design may be more suitable for establishing whether telemedicine is as effective as usual care in the follow-up of leg and foot ulcers.

One could argue that evidence from studies of telemedicine interventions in other disease fields should be used to inform implementation of telemedicine in the follow-up of leg and foot ulcers. For example, telemonitoring approaches have been found therapeutically effective in chronic heart failure and secondary prevention of coronary heart disease [8]. Moreover, telemedicine interventions have been found moderately effective in reducing the risk of disease-related hospitalizations in patients with diabetes and asthma [22,23], although studies were small and conducted over short periods of time. Nevertheless, patients with foot and leg ulcers present a complex group of patients in clinical practice that might require specific adaptations of the telemedicine follow-up. Thus, we argue the need for further trials on these particular patients groups, preferably in separate trials due to somewhat different ulcer aetiologies, standards of care and response to therapy [49].

Safety issues and adverse effects are important aspects to consider when evaluating the effectiveness of telemedicine, including follow –up of patients with leg and foot ulcers. In spite of the promises and benefits that telemedicine is capable of delivering, there are numeral challenges at patient, technical, and legal level. For example, telemedicine may alter the relationship between patients and health professionals compared to face-to-face, in-the-same-room encounters that are typical in usual care settings [52]. Moreover, lack of competence among health professional hinder efficient implementation and might result in adverse events among those receiving care. Another significant concern about telemedicine is that the system must be secured, to prevent unauthorized access to the information. Legal regulations are therefore important but need to balance security without becoming a hurdle to implementation [52]. Safety issues and adverse events were not reported in the included study [48]. These matters are also infrequently reported in systematic reviews of telemedicine interventions [8] and should thus be addressed in future studies.

A key question in evaluating complex telemedicine interventions is whether they are effective in everyday practice [48]. In response to this key issue, we present implications for further research to evaluate the effectiveness of telemedicine interventions in leg and foot ulcers using the EPICOT format [53] (Table 7). We describe a complex intervention aiming for a new service model that incorporates telemedicine, emphasizing clinical outcomes. A second key question is how the intervention works: what are the active ingredients and how are they exerting their effect? [48]. Therefore, it will be important to investigate the impact of the service in both primary and specialist health care using qualitative research methods. Such process evaluation should also explore the experiences of patients and clinicians, and priorities of policy makers in the use of telemedicine.

**Table 7:** **Research recommendations for future studies on the effect of telemedicine follow-up care of leg and foot ulcers based on EPICOT format**

		**Issues to consider**	**Example**
**Core elements**			
E	Evidence	What is the current evidence?	One small study (n = 140) with a non-randomized design conducted in the United States.
P	Population		Patients (>20 years) presenting a leg ulcer or diabetes-related foot ulcer to specialist health care.
I	Intervention		Telemedicine follow-up care provided by municipal primary health care in collaboration with specialist health care
C	Comparison	Placebo, routine care, alternative treatment/management	Care as usual.
O	Outcome	Which clinical or patient related outcomes will the researcher need to measure, improve, influence or accomplish? Which methods of measurement should be used?	Healing time; total number of consultations per person; sequelae directly related to the foot or leg ulcer: infection, hospitalization, and vascular surgery during the study; patient satisfaction with health care; health status and cost utility; the time elapsing before a new ulcer appears, the incidence of amputation and survival.
T	Time stamp	Date of literature search or recommendation	May 16th, 2014.
**Optional elements**			
d	Disease burden		Leg ulcers and diabetes-related foot ulcers are longstanding and costly complications of their underlying diseases and represent challenges for individual people and health care system. Treatment of leg ulcers and diabetic foot ulcers often demands frequent contact with the health care system and may pose a great burden on the patient. According to international guidelines patients with leg or foot ulcers should be referred to specialist foot clinics at an early stage. However, in Norway as well as other European countries many foot ulcer patients are treated a substantial time in primary care with lack of expert nurses and doctors and access to specialist health care, which may be problematic as they may not be using the evidence base sufficiently well to support ulcer healing and patient well-being.
t	Timeliness	Time aspects of core elements:	
		Mean age of the population	67 years
		Duration of the intervention	12 months
		Length of follow-up	3 years
s	Study type	What is the most appropriate study design to address the proposed question	Cluster- randomized controlled trial

## Conclusion

The systematic review assessed whether telemedicine follow-up care of patients with leg and foot ulcers positively affected clinical, behavioral and organizational outcomes compared with traditional follow-up. We only identified one small non-randomized study that met the inclusion criteria, but lacked rigor. Thus the available evidence is too weak to make conclusions about the effectiveness of telemedicine follow-up care of patients with leg and foot ulcers. Larger and more rigorous studies are needed to enable strong conclusions and clinical recommendations to be made.

## Abbreviation

ICT: Information and communication technology
